# 12-month prevalence of osteoporosis in Germany

**DOI:** 10.17886/RKI-GBE-2017-067

**Published:** 2017-10-09

**Authors:** Judith Fuchs, Christa Scheidt-Nave, Ronny Kuhnert

**Affiliations:** Robert Koch Institute, Department of Epidemiology and Health Monitoring, Berlin

**Keywords:** OSTEOPOROSIS, PREVALENCE, ADULTS, HEALTH MONITORING, GERMANY

## Abstract

Osteoporosis is a systemic skeletal disease associated with increased bone fragility, which correspondingly leads to increased bone fractures. In the GEDA 2014/2015-EHIS survey, 7.8% of women and 2.0% of men aged 18 and over reported suffering from osteoporosis during the past 12 months. The share of people reporting osteoporosis increases considerably in the age group 65 and over. Elder women significantly more often report osteoporosis than men. As this was the first time the present indicator for the 12-month prevalence of osteoporosis was evaluated in the context of the European Health Interview Survey (EHIS) 2014/2015, there is no comparative data available.

## Introduction

The Umbrella Organization for Osteology (Dachverband Osteologie) defines osteoporosis as a systemic skeletal disease that is characterised by low bone mass and a microarchitectural deterioriation of bone tissue which results in greater bone fragility [1]. With osteoporosis patients, external causes that would not normally affect a healthy bone can already cause bone fractures (fragility fractures). These fragility fractures most commonly occur in the vertebrae, the upper parts of the thigh bone close to the hip (femoral neck and trochanteric region) as well as the forearm close to the wrist (in particular the distal radius) [2].

Various factors contribute to osteoporosis. There are behavioural risk factors, which persons can influence such as a lack of physical exercise and dietary habits, as well as further risk factors such as underlying diseases and also certain medications. These risks can be reduced by treating the underlying causes or by adapting behaviour. Moreover, there are also non-modifiable factors such as age, female gender and familial predisposition [1].

Osteoporosis has health policy relevance mainly because osteoporosis incidence rates and the consequences of fragility fractures increase with age. Fractures of the thigh bone close to the hip as well as of the vertebrae particularly impact people’s quality of life and their ability to live independently. Unlike fractures of the vertebrae, which often go unrecognised, fractures of the bone close to the hip joint are rarely overlooked and therefore treated in hospital. Hip fracture surgery and subsequent rehabilitation, as well as age-related co- and multi-morbidity issues generate high costs to the health system [3, 4].

## Indicator

The osteoporosis indicator in the GEDA 2014/2015-EHIS health interview survey was calculated using a self-administered paper-based or online questionnaire. Numerous diseases and conditions were surveyed by asking: ‘During the past twelve months have you had any of the following diseases or conditions?’ and: ‘Has a physician ever diagnosed you with any of the following diseases or conditions?’ followed by a list of diseases. Respondents were asked to provide information on whether they had had osteoporosis during the past twelve months. The results were presented stratified by gender, age group and education. Differences between these groups are interpreted as statistically significant if the respective confidence intervals do not overlap.

**GEDA 2014/2015-EHIS**

**Data holder:** Robert Koch Institute

**Aims:** To provide reliable information about the population’s health status, health-related behaviour and health care in Germany, with the possibility of a European comparison

**Method:** Questionnaires completed on paper or online

**Population:** People aged 18 years and above with permanent residency in Germany

**Sampling:** Registry office sample; randomly selected individuals from 301 communities in Germany were invited to participate

**Participants:** 24,016 people (13,144 women; 10,872 men)

**Response rate:** 26.9%

**Study period:** November 2014 - July 2015

**Data protection:** This study was undertaken in strict accordance with the data protection regulations set out in the German Federal Data Protection Act and was approved by the German Federal Commissioner for Data Protection and Freedom of Information. Participation in the study was voluntary. The participants were fully informed about the study’s aims and content, and about data protection. All participants provided written informed consent.

More information in German is available at

www.geda-studie.de

The following analyses are based on the data from 22,344 participants aged 18 and over (12,270 women, 10,074 men) with valid information on having suffered from osteoporosis during the past 12 months. The calculations were carried out using a weighting factor that corrects for deviations within the sample from the German population (as of 31 December 2014) with regard to gender, age, district type and education. District type reflects a particular area’s degree of urbanisation and accounts for the regional distribution found in Germany. The International Standard Classification of Education (ISCED) was used to classify the responses provided on educational level [5]. Lange et al 2017 [6] provides a detailed description of the methodology applied in GEDA 2014/2015-EHIS, as does the article German Health Update: New data for Germany and Europe, which was published in Issue 1/2017 of the Journal of Health Monitoring.

## Results and discussion

7.8% of women and 2.0% men reported having suffered from osteoporosis during the past 12 months (Table 1). The share of people reporting osteoporosis increases considerably with age. Young and middle-aged adults only rarely report osteoporosis. Prevalence in the age group under 45 is below 1% for both genders (Table 1). In the age group 45 to 64, 4.4% of women and 1.9% of men report osteoporosis. Beyond the age of 65 osteoporosis rates increase significantly: nearly one quarter of women (24%) and 5.6% of men are affected. Osteoporosis rates are significantly higher among women in the age groups 45 to 64 and 65 and over than among men.

Osteoporosis prevalence is significantly higher among women aged 45 to 64 with a low-education background (7.2%) than among those with a medium- (4.0%) or high-education (2.9%) background. Low overall rates of osteoporosis in the other age groups allow no conclusions on the significance of levels of education for differences in osteoporosis prevalence in these groups. Possible links between prevalence and education are subject to controversial discussion among researchers [7], and need to be proved by further studies. These will also need to consider changes in the prescription of hormone replacement therapy, which also affects bone structure.

There are no regional differences in osteoporosis prevalence between federal states.

Estimates on the prevalence of osteoporosis vary depending on the type of data collection, sources and the composition of the survey population. Given that guidelines on the diagnosis and treatment of osteoporosis have changed in recent years [1], comparisons of results need to take into account the time period in which data was collected. However, sex differences regarding the prevalence of self-reported osteoporosis, which are described in this Fact sheet, are confirmed by all previous national surveys that have been conducted in Germany [8-11].

Comparability with the results from a previous telephone interview survey conducted by the Robert Koch Institute in 2012 (GEDA 2012) are limited due to differences in posing the question (time frame: ever vs. 12-months; physician diagnosis vs. self-assessment). Due to EHIS regulations, GEDA 2014/2015-EHIS, was conducted using a new system of data collection. The survey for the first time collected data on the 12-month prevalence of diseases and used the results for comparisons between EU countries.

Overall, the new indicator for the prevalence of osteoporosis in Germany reveals the characteristic pattern of a common and age-related chronic disease with a typically higher prevalence among women compared to men. Current recommendations for osteoporosis prophylaxis include regular physical exercise, preventing immobility and falls as well as ensuring a sufficient intake of calcium and vitamin D. Doctors should also assess whether patients take prescribed medications that increase the risk of fractures. Risks-and-benefits analyses can help to establish the usefulness of such medication on a case-to-case basis [1].

## Key statements

- Around 8% of women and 2% of men aged 18 and over reported osteoporosis during the past 12 months.
- The share of people reporting osteoporosis increases considerably with age.
- Women aged 45 and older significantly more often report osteoporosis than men.

## Figures and Tables

**Table 1:** 12-month prevalence of osteoporosis by gender, age and educational level (n=12,270 women; n=10,074 men) Source: GEDA 2014/2015-EHIS

Women	%	(95% CI)	Men	%	(95% CI)
**Women total**	**7.8**	**(7.2-8.5)**	**Men total**	**2.0**	**(1.7-2.4)**
**18-29 Years**	0.3	(0.1-1.1)	**18-29 Years**	0.3	(0.1-0.8)
Low education	-	-	Low education	0.5	(0.1-2.3)
Medium education	0.4	(0.1-1.8)	Medium education	0.3	(0.1-0.9)
High education	-	-	High education	-	-
**30-44 Years**	0.7	(0.4-1.2)	**30-44 Years**	0.5	(0.2-1.0)
Low education	1.8	(0.6-4.8)	Low education	1.2	(0.4-4.2)
Medium education	0.8	(0.4-1.5)	Medium education	0.5	(0.2-1.3)
High education	-	-	High education	0.2	(0.0-0.8)
**45-64 Years**	4.4	(3.7-5.1)	**45-64 Years**	1.9	(1.4-2.6)
Low education	7.2	(5.2-9.9)	Low education	2.7	(1.3-5.7)
Medium education	4.0	(3.2-5.0)	Medium education	2.4	(1.7-3.4)
High education	2.9	(2.0-4.2)	High education	0.7	(0.4-1.3)
**≥ 65 Years**	24.0	(21.9-26.2)	**≥ 65 Years**	5.6	(4.5-6.9)
Low education	25.9	(22.9-29.1)	Low education	9.0	(6.3-12.8)
Medium education	23.4	(20.6-26.4)	Medium education	5.3	(3.9-7.2)
High education	18.2	(14.0-23.2)	High education	4.6	(3.2-6.5)
**Total (women and men)**	**5.0**	**(4.6-5.4)**	**Total (women and men)**	**5.0**	**(4.6-5.4)**

CI=Confidence interval
